# Reverse total shoulder replacement versus anatomical total shoulder replacement for osteoarthritis: population based cohort study using data from the National Joint Registry and Hospital Episode Statistics for England

**DOI:** 10.1136/bmj-2023-077939

**Published:** 2024-04-30

**Authors:** Epaminondas Markos Valsamis, Albert Prats-Uribe, Ian Koblbauer, Sophie Cole, Adrian Sayers, Michael R Whitehouse, Gillian Coward, Gary S Collins, Rafael Pinedo-Villanueva, Daniel Prieto-Alhambra, Jonathan L Rees

**Affiliations:** 1 Botnar Research Centre, Nuffield Department of Orthopaedics, Rheumatology and Musculoskeletal Sciences, University of Oxford, Oxford, UK; 2 NIHR Oxford Biomedical Research Centre, Oxford, UK; 3 Health Data Sciences, Nuffield Department of Orthopaedics, Rheumatology and Musculoskeletal Sciences, University of Oxford, Oxford, UK; 4 Centre for Statistics in Medicine, Nuffield Department of Orthopaedics, Rheumatology and Musculoskeletal Sciences, University of Oxford, Oxford, UK; 5 Musculoskeletal Research Unit, Bristol Medical School, Southmead Hospital, University of Bristol, Bristol, UK; 6 NIHR Bristol Biomedical Research Centre, Bristol, UK; 7 National Joint Registry Research Committee, National Joint Registry, UK

## Abstract

**Objectives:**

To answer a national research priority by comparing the risk-benefit and costs associated with reverse total shoulder replacement (RTSR) and anatomical total shoulder replacement (TSR) in patients having elective primary shoulder replacement for osteoarthritis.

**Design:**

Population based cohort study using data from the National Joint Registry and Hospital Episode Statistics for England.

**Setting:**

Public hospitals and publicly funded procedures at private hospitals in England, 2012-20.

**Participants:**

Adults aged 60 years or older who underwent RTSR or TSR for osteoarthritis with intact rotator cuff tendons. Patients were identified from the National Joint Registry and linked to NHS Hospital Episode Statistics and civil registration mortality data. Propensity score matching and inverse probability of treatment weighting were used to balance the study groups.

**Main outcome measures:**

The main outcome measure was revision surgery. Secondary outcome measures included serious adverse events within 90 days, reoperations within 12 months, prolonged hospital stay (more than three nights), change in Oxford Shoulder Score (preoperative to six month postoperative), and lifetime costs to the healthcare service.

**Results:**

The propensity score matched population comprised 7124 RTSR or TSR procedures (126 were revised), and the inverse probability of treatment weighted population comprised 12 968 procedures (294 were revised) with a maximum follow-up of 8.75 years. RTSR had a reduced hazard ratio of revision in the first three years (hazard ratio local minimum 0.33, 95% confidence interval 0.18 to 0.59) with no clinically important difference in revision-free restricted mean survival time, and a reduced relative risk of reoperations at 12 months (odds ratio 0.45, 95% confidence interval 0.25 to 0.83) with an absolute risk difference of −0.51% (95% confidence interval −0.89 to −0.13). Serious adverse events and prolonged hospital stay risks, change in Oxford Shoulder Score, and modelled mean lifetime costs were similar. Outcomes remained consistent after weighting.

**Conclusions:**

This study’s findings provide reassurance that RTSR is an acceptable alternative to TSR for patients aged 60 years or older with osteoarthritis and intact rotator cuff tendons. Despite a significant difference in the risk profiles of revision surgery over time, no statistically significant and clinically important differences between RTSR and TSR were found in terms of long term revision surgery, serious adverse events, reoperations, prolonged hospital stay, or lifetime healthcare costs.

## Introduction

**Fig 1:**
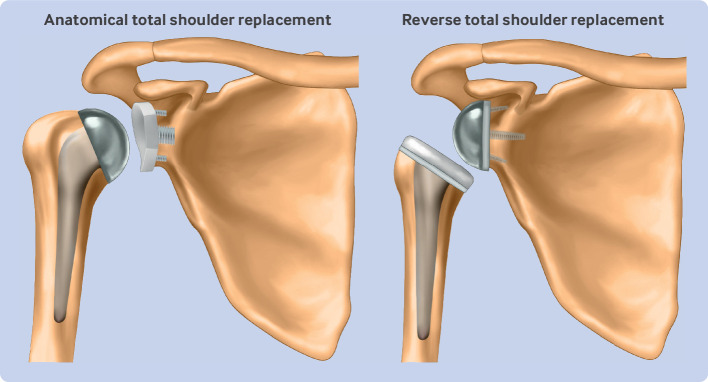
Anatomical and reverse total shoulder replacements. Anatomical total shoulder replacement—prosthetic ball and socket replacement that matches normal ball and socket anatomy of shoulder joint. Reverse total shoulder replacement—prosthetic ball and socket replacement that reverses normal ball and socket anatomy of shoulder joint

Shoulder replacement surgery is an effective treatment option for end stage shoulder arthritis, and is rising in incidence internationally.^1^ ^2^ The use of reverse total shoulder replacement (RTSR), initially developed for rotator cuff arthropathy, has now expanded globally across different surgical indications, including osteoarthritis with an intact rotator cuff, a common condition traditionally treated with an anatomical total shoulder replacement (TSR; fig 1).^3^ ^4^ ^5^ ^6^ This shift in practice is growing despite a lack of supporting evidence, a concern highlighted by the National Institute for Health and Care Excellence and patients, carers, and clinicians during a James Lind Alliance Priority Setting Partnership.^7^ ^8^

Healthcare agencies like the National Institute for Health and Care Research have now funded randomised controlled trials to address this lack of evidence.^9^ However, such trials can take more than five years to complete and usually only report two year clinical outcomes. Additionally, no guarantees of completion exist because of the unique challenges facing surgical trials, such as prolonged recruitment, changes in healthcare pathways, and shifts in community equipoise.^10^ ^11^ Importantly, randomised controlled trials in this context cannot assess long term implant survival for many years, while the smaller sample sizes are usually insufficient to evaluate the relatively low number of serious adverse events seen in elective orthopaedics. The use of routinely collected data, particularly from large national joint registries, can potentially expedite answers at a fraction of the cost.^12^ A recent study highlighted the value of observational studies in emulating trials, emphasising external validity in real world populations.^13^ Examples in orthopaedics include successful emulation of the TOPKAT (total or partial knee arthroplasty) trial comparing different types of knee replacement.^14^ This has improved the generalisability of the TOPKAT trial results and their acceptability to the surgical community.

With the commissioning of surgical randomised controlled trials in response to the global increase in use of RTSR for osteoarthritis, our objective was to use modern epidemiological methods and national datasets to provide more rapid high quality evidence to the international community on safety and revision surgery. Such evidence will complement future randomised controlled trial results that will examine in more detail patient functional outcomes and cost effectiveness. The aim of this study was to examine the clinical effectiveness and economic implications of using RSTR over the anatomical TSR when treating patients aged 60 years or older with osteoarthritis and an intact rotator cuff.

## Methods

### Study design and data sources

Population based prospective cohort study using linked, routinely collected data for shoulder replacements undertaken at public hospitals, and publicly funded procedures in private hospitals in England from 1 April 2012 to 31 December 2020. Data from the National Joint Registry of England were linked to the NHS Hospital Episode Statistics database and to civil registration mortality data. Data submission to the National Joint Registry is mandatory for all shoulder replacements occurring at public and private hospitals, and includes patient, surgeon, and operation details. The Hospital Episode Statistics admitted patient care database records all inpatient and day case activity in public hospitals and publicly funded procedures in private hospitals in England, and includes demographic data, medical diagnoses, procedural and administrative information. Hospital Episode Statistics data are used for the accurate reimbursement of NHS providers for their activities.

### Selection criteria

All patients aged 60 years or older having an elective primary shoulder replacement for osteoarthritis with an intact rotator cuff were eligible for inclusion in the study. Patients were included if they received a TSR or an RTSR. The intact condition of the rotator cuff is determined by the operating surgeon at the time of surgery and recorded by the National Joint Registry through a mandatory minimum dataset form.^15^ Procedures where the rotator cuff was recorded as absent or torn were excluded. The patient group in this study closely aligns with that in the commissioned RAPSODI trial (reverse or anatomical replacement for painful shoulder osteoarthritis: differences between interventions, ISRCTN 12216466), both of which rely on the intraoperative assessment of the rotator cuff.^9^ We excluded patients with inconsistent surgical histories (ie, revision or death predated their primary procedure) and duplicates. The unit of analysis was considered the procedure rather than the patient, so a patient’s left and right sided shoulder replacements would appear as separate observations.

### Clinical outcomes

The primary outcome was revision surgery at any time point (representing implant survival). The National Joint Registry defines revision as a procedure that involves adding, removing, or modifying one or more components of a joint prosthesis, and is the most commonly used metric for assessing the success of joint replacement surgery.^5^

Secondary outcomes included serious adverse events occurring within 90 days of surgery, reoperations within 12 months of surgery, prolonged hospital stay, and change in Oxford Shoulder Score (preoperative to six month postoperative). Serious adverse events were defined as admissions to hospital owing to any of the following medical complications, identified using ICD-10 (International Classification of Diseases, 10th revision) codes: pulmonary embolism, myocardial infarction, lower respiratory tract infection, acute kidney injury, urinary tract infection, cerebrovascular events, and all cause death.^16^ Repeat surgery on the same shoulder within 12 months of the primary procedure that did not meet the criteria for revision, and that occurred on a separate occasion to any revision surgery, was termed a reoperation and was identified from relevant OPCS-4 (Office of Population Censuses and Surveys classification of surgical operations and procedures, fourth revision) codes (see supplementary tables S1-S3). After consultation with patient representatives from a patient advisory panel for this study, prolonged hospital stay was defined as an inpatient duration greater than three nights from the date of the primary procedure. The preoperative and six month postoperative Oxford Shoulder Score (a shoulder specific questionnaire—from a minimum score of 0 to a maximum score of 48), a patient reported outcome measure, collected by the National Joint Registry, was available for a subset of patients.

### Costs

Hospital costs associated with the primary (including serious adverse events and reoperations) and revision (including serious adverse events) procedures were estimated using healthcare resource group codes (which classify hospital activity according to resource use), length of hospital stay, and unbundled healthcare resource group codes (individually priced consumable elements).^17^ Healthcare resource group codes were valued using 2020-21 NHS reference costs. These costs represent the average cost to the NHS of providing a defined service in a given financial year. The calculations were used to generate the reimbursement value of each primary and revision procedure to the hospital provider based on the National Reimbursement System.^18^ ^19^

### Statistical analysis

#### Clinical analysis

Propensity scores were generated using logistic regression and represent the probability of a patient receiving an RTSR, as opposed to a TSR, based on the following covariates: age, sex, American Society of Anaesthesiologists grade, thromboprophylaxis, previous shoulder surgery, rural or urban residence, socioeconomic deprivation (area level index of multiple deprivation), operation funding, Charlson comorbidity index, obesity, past medical history of gastrointestinal, mental health, respiratory, circulatory, metabolic, neurological, and urinary tract problems, and health hazards. One-to-one propensity score matching using callipers of width equal to 0.2 of the standard deviation of the logit of the propensity score was used to enable estimation of the average treatment effect on the treated population.^20^ ^21^ Inverse probability of treatment weighting for participants on the common support of propensity scores was used for the average treatment effect on the total population.^22^ The combined evaluation of average treatment effect on the treated and average treatment effect on the total population is essential for capturing any heterogeneity across different patient subgroups: average treatment effect on the treated population focuses on the effects within the treatment group, reflecting scenarios where specific patient characteristics influence treatment allocation, while average treatment effect on the total population captures effects on the treated and control groups, and is a key consideration when formulating potentially universal healthcare policies.^23^

Covariate balance was assessed before and after matching and weighting, with an absolute standardised mean difference of 10% or more considered indicative of imbalance.^24^ Additionally, negative control outcomes were analysed to test for unmeasured confounding, and included hip fracture, vertebral fracture, hernia and acute upper respiratory tract infection within a year of surgery, chosen for their lack of any plausible association with procedure type.^25^

Relative and absolute treatment effects were estimated for each outcome after matching and weighting.^26^ Flexible parametric survival models (using restricted cubic splines to allow for modelling nonlinearity in the baseline hazard function) including treatment allocation as a time varying covariate were used for revision, while logistic regression was used for the binary secondary outcomes. Robust variance estimation was used to account for the clustering within matched sets and the weighted nature of the samples.^27^ ^28^ An additional analysis was undertaken using linear regression to estimate the treatment effect on the change (preoperative to six month postoperative) in Oxford Shoulder Score in a subset of patients with non-missing patient reported outcomes.^29^

#### Cost analysis

**Fig 2:**
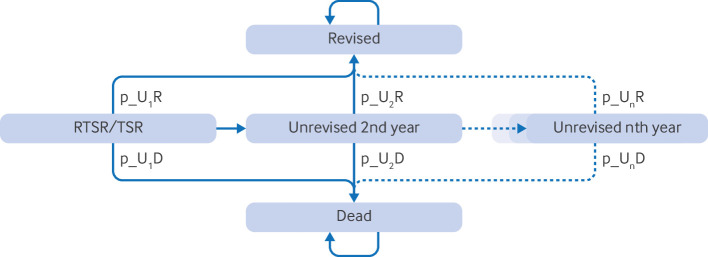
Cohort state transition (Markov) model for cost analysis. p_U_n_R=time dependent (nth cycle) transition probability from unrevised state to revised state; p_U_n_D=time dependent (nth cycle) transition probability from unrevised state to dead state (unrevised state consisted of tunnel states, accounting for time dependency; revised and dead states are absorbing states with no transitions out of them); RTSR=reverse total shoulder replacement; TSR=anatomical total shoulder replacement

A lifetime Markov model provided the framework for the cost analysis, with patients passing from clinically and economically important health states over annual cycles (fig 2).^30^ Parametric models were specified for each treatment group to estimate the risk of revision and death, informing the model’s time dependent transition probabilities.^31^ Model estimates were used to extrapolate the risk of revision and death past the study period. A sensitivity analysis was performed where the risk of death returns to that specified by age and sex specific UK life tables after the period of follow-up.^32^ Future costs were discounted at the established annual rate of 3.5%, and the effect of varying the discount rate was reported.^33^

This evaluation was undertaken from a healthcare system perspective, so the expected (mean) inpatient costs incurred by the health system were estimated for each treatment group. The effect of parameter uncertainty was assessed using probabilistic sensitivity analysis, with the model’s input parameters assigned from probability distributions for 10 000 Monte Carlo simulations. Gamma distributions were used for costs while Cholesky decompositions were used to provide correlated draws from multivariate normal distributions, which then informed the transition probabilities (supplementary figures S6-S10, tables S5-S12).^31^ Stata V.16.1 was used for the statistical analyses; R software was used for the cost analysis.^34^ ^35^

#### Sensitivity analyses

For the first sensitivity analysis, we limited inclusion to procedures performed by surgeons who carried out 11 or more combined trauma and elective shoulder replacement procedures in the year preceding the surgery. The rationale for this threshold was derived from a previously reported volume effect threshold of 10.4 procedures per year.^36^ The second sensitivity analysis limited inclusion to procedures performed by surgeons considered to have balanced practice between RTSR and TSR for elective surgery. Practice was considered balanced when surgeons completed no more than 80% of TSR or RTSR as a proportion of the sum of these two procedures across their elective practice in the preceding year. The threshold of 80% was pragmatically determined to ensure reasonably balanced practice while not overly restricting the sample size available. This allowed the calculated proportions to vary dynamically over time for each surgeon, restricting the sensitivity analysis to procedures undertaken by surgeons at times when their caseload reflected balanced practice.

#### Missing data

**Fig 3:**
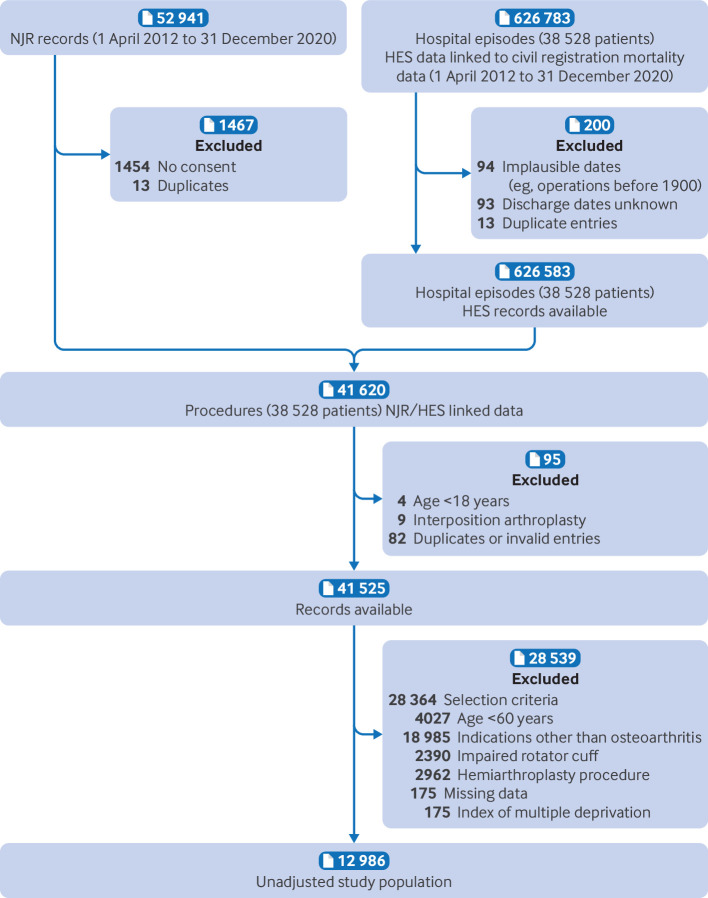
Data flowchart for linked National Joint Registry (NJR), Hospital Episode Statistics (HES), and civil registration mortality dataset

Index of multiple deprivation data were missing for 175 procedures (1.3%), so these were excluded, and a complete case analysis undertaken (fig 3). The preoperative and postoperative Oxford Shoulder Scores were missing for 7903 (61%) and 6448 (50%) procedures, respectively, or were considered invalid (based on accepted questionnaire completion timeframes) for an additional 1523 (12%) and 1696 (13%) procedures, respectively.^5^ Simple mean imputation was used for partially completed Oxford Shoulder Score questionnaires (24%) when no more than two of 12 questions were left blank, as per the instrument’s guidelines.^29^ A total of 1321 (10%) valid, paired preoperative and postoperative Oxford Shoulder Scores remained. Despite largely balanced covariates between questionnaire responders (non-missing) and non-responders (missing), the potential for missing data bias precluded a cost effectiveness analysis in this study, and analysis of patient reported outcomes was excluded from the sensitivity analyses (supplementary table S14).

### Patient and public involvement

One of the top 10 research uncertainties identified by patients and clinicians from the 2015 James Lind Alliance Priority Setting Partnership on shoulder surgery specifically addressed the comparative effectiveness between different shoulder replacement types.^7^ Patient representatives sit on the committee structure of the National Joint Registry. This study’s patient advisory panel also included a patient representative from the National Institute for Health and Care Excellence. Additionally, a patient representative from the National Joint Registry coauthored this study. Our patient representatives defined a hospital stay greater than three nights as prolonged because going home soon after surgery is an important outcome for them.

## Results

### Study population

The study population comprised 12 986 elective primary shoulder replacement procedures in 11 961 patients (fig 3). After propensity score matching (n=7124; TSR: 3562, RTSR: 3562) and adjustment for inverse probability of treatment weighting (n=12 968; TSR: 9393, RTSR: 3575), all covariates were well balanced (table 1).

**Table 1:** Baseline characteristics of study patients

Characteristic	Unadjusted study population (n=12 986)*				Matched study population (n=7124)†				IPTW study population (n=12 968)†		
	TSR (n=9410)	RTSR (n=3576)	ASMD (%)		TSR (n=3562)	RTSR (n=3562)	ASMD (%)		TSR (n=9393)	RTSR (n=3575)	ASMD (%)
Age (years), mean (SD)	72.5 (6.7)	77.1 (6.4)	70.8		76.9 (6.2)	77.0 (6.3)	2.6		73.8 (7.0)	73.9 (6.7)	2.1
Sex											
Male	2668 (28.4)	804 (22.5)	13.5		23.0	22.5	1.0		26.7	26.8	0.1
Female	6742 (71.7)	2772 (77.5)	13.5		77.0	77.5	1.0		73.3	73.2	0.1
ASA grade											
1	663 (7.1)	124 (3.5)	16.1		3.7	3.5	1.1		6.0	6.3	1.1
2	6549 (69.6)	2260 (63.2)	13.6		64.0	63.4	1.3		67.6	66.8	1.8
3	2163 (23.0)	1155 (32.3)	20.9		31.5	32.1	1.4		25.8	26.4	1.4
4-5	35 (0.4)	37 (1.0)	7.9		0.8	1.0	1.8		0.6	0.6	0.2
Operation funding											
Public (NHS)	9242 (98.2)	3526 (98.6)	3.1		98.6	98.6	0.0		98.3	98.3	0.1
Private	168 (1.8)	50 (1.4)	3.1		1.4	1.4	0.0		1.7	1.7	0.1
Previous surgery											
Yes	8607 (91.5)	3287 (91.9)	1.6		91.7	91.9	0.8		91.6	92.0	1.6
No	803 (8.5)	289 (8.1)	1.6		8.3	8.1	0.8		8.4	8.0	1.6
Thromboprophylaxis											
None	101 (1.1)	30 (0.8)	2.4		0.8	0.8	0.0		1.0	1.1	1.2
Chemical	149 (1.6)	85 (2.4)	5.7		2.0	2.3	2.1		1.8	1.8	0.3
Mechanical	2436 (25.9)	624 (17.5)	20.6		18.4	17.5	2.3		23.6	24.4	2.0
Chemical and mechanical	6724 (71.5)	2837 (79.3)	18.4		78.8	79.4	1.4		73.7	72.6	2.3
IMD group											
1 (most deprived)	434 (4.6)	190 (5.3)	3.2		5.2	5.3	0.5		4.7	4.6	0.5
2	569 (6.1)	243 (6.8)	3.1		6.6	6.7	0.6		6.3	6.5	0.8
3	683 (7.3)	277 (7.8)	1.9		7.5	7.7	0.8		7.4	7.2	0.8
4	824 (8.8)	290 (8.1)	2.3		8.3	8.1	0.4		8.6	8.5	0.5
5	1040 (11.1)	341 (9.5)	5.0		9.7	9.5	0.5		10.6	10.8	0.6
6	1184 (12.6)	456 (12.8)	0.5		13.0	12.8	0.6		12.7	12.4	0.9
7	1222 (13.0)	449 (12.6)	1.3		12.0	12.5	1.5		12.8	12.7	0.5
8	1247 (13.3)	455 (12.7)	1.6		12.5	12.8	0.8		13.1	12.7	1.1
9	1129 (12.0)	412 (11.5)	1.5		12.0	11.6	1.5		11.9	12.3	1.2
10 (least deprived)	1078 (11.5)	463 (13.0)	4.6		13.3	12.9	1.0		11.9	12.4	1.6
Rural or urban residence											
Urban—sparse	34 (0.4)	12 (0.3)	0.4		0.4	0.3	0.9		0.4	0.4	0.2
Town and fringe—sparse	77 (0.8)	17 (0.5)	4.3		0.6	0.5	2.3		0.7	0.6	1.2
Village—sparse	129 (1.4)	21 (0.6)	8.0		0.4	0.6	3.3		1.1	1.1	0.2
Hamlet—sparse	82 (0.9)	15 (0.4)	5.6		0.4	0.4	0.4		0.7	0.8	0.9
Urban—less sparse	6389 (67.9)	2572 (71.9)	8.8		72.1	71.8	0.6		69.1	69.6	1.2
Town and fringe—less sparse	1214 (12.9)	455 (12.7)	0.5		13.4	12.8	1.9		12.9	12.7	0.7
Village—less sparse	1035 (11.0)	329 (9.2)	6.0		8.9	9.2	1.3		10.5	10.2	0.9
Hamlet—less sparse	450 (4.8)	155 (4.3)	2.1		3.8	4.4	2.5		4.7	4.6	0.3
Gastrointestinal diseases‡											
Yes	3321 (35.3)	1462 (40.9)	11.5		39.4	40.7	2.6		36.9	36.9	0.1
No	6089 (64.7)	2114 (59.1)	11.5		60.6	59.3	2.6		63.1	63.1	0.1
Mental health diseases‡											
Yes	1713 (18.2)	684 (19.1)	2.4		18.4	19.1	1.9		18.4	17.9	1.5
No	7697 (81.8)	2892 (80.9)	2.4		81.6	80.9	1.9		81.6	82.1	1.5
Respiratory diseases‡											
Yes	2281 (24.2)	971 (27.2)	6.7		25.6	27.1	3.4		25.0	25.0	0.0
No	7129 (75.8)	2605 (72.9)	6.7		74.4	72.9	3.4		75.0	75.0	0.0
Circulatory diseases‡											
Yes	6348 (67.5)	2626 (73.4)	13.1		73.5	73.4	0.3		69.3	69.0	0.7
No	3062 (32.5)	950 (26.6)	13.1		26.5	26.6	0.3		30.7	31.0	0.7
Metabolic diseases‡											
Yes	3489 (37.1)	1561 (43.7)	13.4		43.8	43.6	0.5		39.1	39.5	0.7
No	5921 (62.9)	2015 (56.4)	13.4		56.2	56.4	0.5		60.9	60.5	0.7
Neurological diseases‡											
Yes	1264 (13.4)	521 (14.6)	3.3		14.5	14.5	0.2		13.9	14.1	0.7
No	8146 (86.6)	3055 (85.4)	3.3		85.5	85.5	0.2		86.1	85.9	0.7
Urinary tract diseases‡											
Yes	1660 (17.6)	871 (24.4)	16.5		23.7	24.1	1.1		19.5	19.1	0.9
No	7750 (82.4)	2705 (75.6)	16.5		76.3	75.9	1.1		80.5	80.9	0.9
Health hazards‡											
Yes	150 (1.6)	92 (2.6)	6.9		2.6	2.6	0.2		1.9	1.9	0.3
No	9260 (98.4)	3484 (97.4)	6.9		97.4	97.4	0.2		98.1	98.1	0.3
Obesity‡											
Yes	2406 (25.6)	908 (25.4)	0.4		25.0	25.4	0.8		25.4	24.9	1.3
No	7004 (74.4)	2668 (74.6)	0.4		75.0	74.6	0.8		74.6	75.1	1.3
Charlson comorbidity index§											
0	4231 (45.0)	1295 (36.2)	17.9		36.8	36.4	0.9		42.4	41.7	1.3
1	2322 (24.7)	850 (23.8)	2.1		24.6	23.8	2.0		24.5	24.6	0.3
≥2	2857 (30.4)	1431 (40.0)	20.3		38.6	39.9	2.6		33.1	33.7	1.1

ASA=American Society of Anaesthesiologists; ASMD=absolute standardised mean difference; IMD=index of multiple deprivation; IPTW=inverse probability of treatment weighting; RTSR=reverse total shoulder replacement; TSR=anatomical total shoulder replacement.

* Data presented as number (percentage) of procedures.

† Data presented as percentage of procedures.

‡ Comorbidities identified using international classification of diseases (10th revision) codes in Hospital Episode Statistics data (supplementary table S3).

§ Charlson comorbidity index calculated using the “charlson” package in Stata.

### Clinical analysis

#### Primary outcome

In the matched cohort there were 126 revisions (1.8%; TSR: 85, RTSR: 41) with a maximum follow-up of 8.75 years and a total of 24 353 years of observation time (TSR: 14 332, RTSR: 10 021). In the weighted cohort there were 294 revisions (2.3%; TSR: 253, RTSR: 41) with a maximum follow-up of 8.75 years and a total of 47 886 years of observation time (TSR: 37 842, RTSR: 10 044).

**Fig 4:**
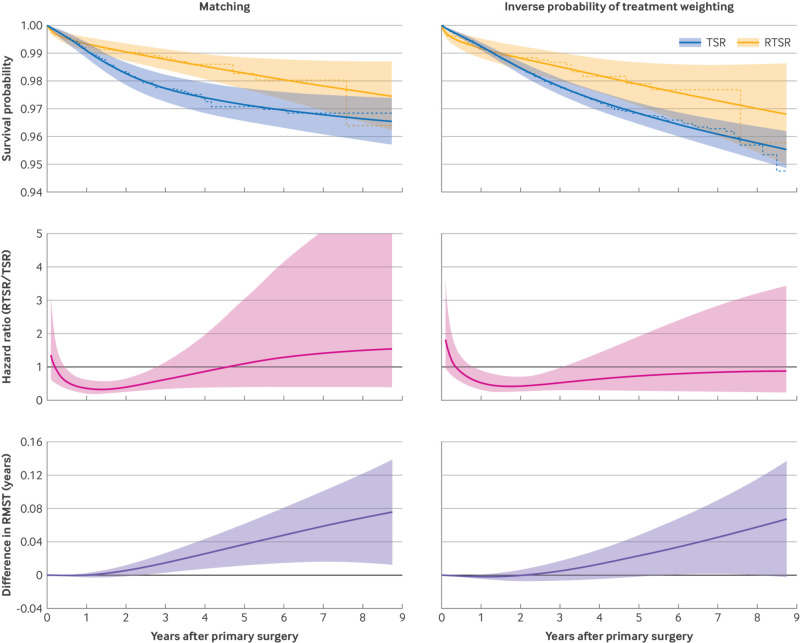
Treatment effects for primary outcome of revision using flexible parametric survival models. Upper panel shows survival probability for the two procedure types overlying Kaplan-Meier curves. Middle panel shows relative risk: time varying hazard ratio of RTSR with reference to TSR. Bottom panel shows absolute risk difference in RMST (RTSR − TSR). RMST=restricted mean survival time; RTSR=reverse total shoulder replacement; TSR=anatomical total shoulder replacement

For the matched and weighted cohorts, the survival curves for RTSR and TSR showed a similar shape with an early (up to one year) increased revision risk for RTSR, followed by an increased revision risk for TSR thereafter (fig 4). However, the confidence intervals for the survival curves overlap throughout (apart from a period at around three years in the matched cohort), indicating a similar longer term (more than eight year) survival probability.

For the matched cohort, flexible parametric model survival analysis showed an immediate non-significant postoperative hazard ratio of 1.39 (95% confidence interval (CI) 0.61 to 3.17) for revision with RTSR, indicating a nearly 40% increased risk of revision in the immediate postoperative period compared with TSR, after which there was a significantly reduced risk until nearly three years (hazard ratio local minimum 0.33, 95% CI 0.18 to 0.59). Similarly, for the weighted cohort, the immediate non-significant postoperative hazard ratio was 1.89 (0.97 to 3.69), followed by a significantly reduced risk until three years (hazard ratio local minimum 0.42, 0.25 to 0.73). The estimated hazard ratio after three years differs between the matched and weighted cohorts, increasing above one or staying below one, respectively, but the confidence intervals cross one throughout, indicating no significant difference. The widened confidence intervals and differing hazard ratio estimates after three years can be attributed to the reducing sample size of patients who remained at risk for longer time periods, but the fact the confidence intervals cross one suggests no statistically significant difference at these longer time periods.

Restricted mean survival time represents the area under the survival curve up to a specific time point.^37^ For the matched cohort, the difference in restricted mean survival time was significant after 2.5 years, and it reached a maximum of 0.076 years at 8.75 years of follow-up (corresponding to a difference of 28 days of revision-free survival favouring RTSR). For the weighted cohort, the difference in restricted mean survival time was significant between 5.9 and 8.0 years, not before or after that period, and it reached a maximum of 0.067 years at 8.75 years of follow-up (corresponding to a difference of 24 days favouring RTSR).

Therefore, the differences in the shapes of the survival curves can be explained by changes in the relative hazard ratio over time (favouring RTSR between 0.5 and 3 years), amounting to a statistically significant but not clinically important absolute risk difference (less than 30 days’ difference of revision free survival by over eight years of follow-up). There was no statistically significant and clinically important difference in the relative risk or absolute risk difference by the end of the study period for either cohort.

#### Secondary outcomes

**Fig 5:**
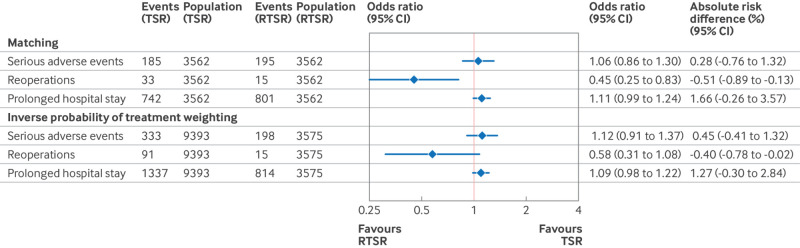
Treatment effects for secondary outcomes using logistic regression models. RTSR=reverse total shoulder replacement; TSR=anatomical total shoulder replacement. “Events” represents the positive count of the outcome measure while “population” represents the at-risk cohort. Absolute risk difference=RTSR − TSR

In the matched cohort, after RTSR, the relative risk of reoperations within 12 months was half (odds ratio 0.45, 95% CI 0.25 to 0.83), with an absolute risk difference of −0.51% (95% CI −0.89 to −0.13), although there was no significant difference in relative risk in the weighted cohort (0.58, 0.31 to 1.08; fig 5). While statistically significant, an absolute risk difference of around 1 in 200 is likely to be of no clinical importance. There was no statistically significant difference in the absolute or relative risk of serious adverse events or prolonged hospital stay for the matched or weighted cohorts.

Results were consistent in both sensitivity analyses (supplementary figures S2-S4). Treatment effects for negative control outcomes were not statistically significant and centred around one, suggesting no residual confounding (supplementary figure S1).

While the Oxford Shoulder Score was not included when generating propensity scores, in the matched and weighted study populations, the absolute standardised mean difference of the preoperative Oxford Shoulder Score was 8.0% and 7.2%, respectively. In the matched population there were 709 (10%) non-missing, valid, paired preoperative and six month postoperative scores with an estimated linear regression coefficient of −0.21 (95% CI −1.70 to 1.29), indicating a non-significant difference in the change in score in favour of TSR. In the weighted population there were 1319 (10%) non-missing, valid, paired scores with an estimated linear regression coefficient of 0.99 (−0.53 to 2.51), indicating a non-significant difference in favour of RTSR (supplementary figure S11, table S13).

### Cost analysis

**Fig 6:**
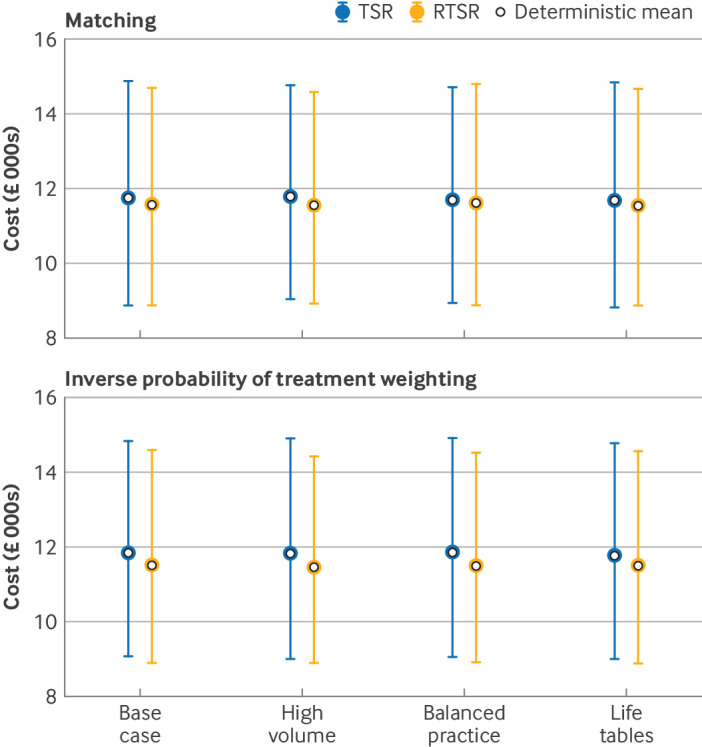
Cost analysis results. Probabilistic sensitivity analysis results after 10 000 Monte Carlo simulations shown with 95% confidence intervals, together with deterministic mean results. RTSR=reverse total shoulder replacement; TSR=anatomical total shoulder replacement

A total of 12 549 procedures (96.6%) successfully generated a valid healthcare resource group code and were valued using NHS reference costs. While the mean estimated lifetime costs of TSR were slightly higher than those of RTSR for the base case and sensitivity analyses, the overlapping confidence intervals derived from the probabilistic sensitivity analysis confirm there is no significant difference between the two procedures (fig 6). Results were consistent after matching and weighting, and were unaffected by changes in discount rate (supplementary figure S10).

## Discussion

This study used national, linked, prospective routinely collected registry and hospital data to compare the clinical outcomes and cost implications between RTSR and TSR in patients aged 60 years or older with osteoarthritis and an intact rotator cuff. The two procedure types had a significantly different revision risk profile over time, with RTSR being associated with a reduced (under half) risk of early revision surgery until three years. However, there was no clinically important absolute risk difference found throughout the study period. Despite a statistically significant difference in the relative risk of reoperations within 12 months favouring RTSR in the matched cohort, this did not amount to a clinically important absolute risk difference, and there was no significant difference in the weighted cohort. There was no statistically significant difference for the outcomes of serious adverse events within 90 days or prolonged hospital stay, and estimated lifetime costs to the healthcare service were similar. Only a limited analysis of patient reported outcomes was possible owing to completeness of the collected data, but this did not suggest any statistically significant difference in the change in Oxford Shoulder Score.

### Strengths and weaknesses of this study

The main strengths of this study lie in its large sample size and robust methods, including clinically relevant sensitivity analyses. While randomised controlled trials emphasise the internal validity of claims about causality, observational studies emphasise external validity in real word populations.^12^ ^13^ This observational study provides real world evidence by delineating treatment effects within the RTSR recipient population (average treatment effect on the treated population) and across the entire eligible demographic (average treatment effect on the total population). Compared with randomised controlled trials, observational studies such as this one are often more cost efficient and can be conducted in a much shorter timeframe. Observational studies provide high level evidence and generalisable results when randomised controlled trial data are lacking, and can complement findings from randomised controlled trials. Moreover, observational studies can better reflect real world surgical practice, enhancing their value in informing clinical decisions.

While randomised controlled trials are well placed to report differences in patient reported outcomes, their limited sample size constrains their ability to capture the less common outcomes in elective orthopaedic surgery, such as serious adverse events including all cause death. In contrast, the substantial cohort size in our study provides a more robust framework for detecting these infrequent events. Furthermore, our study's longer follow-up period and larger sample size enhance our study’s ability to compare the risk of revision surgery—an outcome that might not be adequately captured within the initial short term follow-up periods seen in randomised controlled trials. The routine collection of registry and hospital data also enables subsequent reanalyses to generate results with even larger sample sizes and longer follow-up in the future.

The main limitation of this study was the absence of a complete dataset for patient reported outcome measures. While we undertook an analysis of patient reported outcomes on a small subset of the study population for which data were available, those results need to be interpreted with caution owing to the risk of reporting bias. Results from trials like RAPSODI on patient reported outcomes will provide more complete information, though based on the observations in this study, we expect there will be no clinically meaningful difference in scores found. The incomplete dataset also precluded a cost effectiveness analysis, which is the accepted standard for health economic evaluations required for national treatment decision making in many countries. Despite adjusting for several clinically relevant variables, being an observational study, the possibility of residual confounding remains. The National Joint Registry has only recently started to collect information on preoperative glenoid morphology and so this variable was unavailable in our dataset. Finally, healthcare resource group codes do not distinguish between the different implants, meaning there might be uncaptured cost differences between procedure types from a hospital provider perspective.

### Comparison with other studies

The debate surrounding the choice between RTSR and TSR for patients with osteoarthritis and an intact rotator cuff has represented a growing research uncertainty.^8^ ^38^ ^39^ One of the more common indications for revision of a TSR in this patient population includes secondary postoperative rotator cuff failure, a complication leading to further surgery that could be avoided with RTSR as the first line option.^40^ This reasoning might partly account for the observed shift to increasing use of RTSR in this patient cohort in international practice. However, robust high quality evidence to substantiate this trend has been lacking.^8^ ^41^ ^42^ ^43^ ^44^ A Cochrane review reported a lack of sufficient evidence to sufficiently compare TSR and RTSR, while a recent systematic review and meta-analysis identified just six small observational studies comparing these two procedures, with less than 150 patients in any one study.^38^ ^45^ A more recent study used a propensity score matched cohort of 370 patients per group. Despite an average follow-up of less than two years, it reported similar patient reported outcomes and revision rates, but an increased risk of adverse events after TSR.^39^ However, the study is not exempt from methodological weaknesses. A limited selection of confounders was considered and covariate balance metrics were inadequately reported. Furthermore, that study treated revision as a binary outcome variable (leading to information loss) and its authors acknowledge the lack of power to detect differences in the risk of revision.^46^ The definition of adverse events was not clear, and the study only presented p values from t tests without calculating relative or absolute risk. One study from the Australian Orthopaedic Association National Joint Replacement Registry compared a selected subset of TSR with RTSR to investigate any differences in the risk of revision surgery up to four years after primary surgery for osteoarthritis, after adjusting for age, gender, American Society of Anaesthesiologists score, and body mass index.^47^ Their study revealed similar initial survival curves where RTSR had a higher risk of revision in the first year, followed by a reduced risk thereafter, although only the first three months showed a significant difference. However, the authors were unable to restrict the analysis to patients with an intact rotator cuff as this variable is not recorded in the Australian Orthopaedic Association National Joint Replacement Registry, meaning that TSR might have been compared with an RTSR patient group with impaired or torn rotator cuffs.

### Meaning of the study, unanswered questions, and future research

In response to the rapid rise of offering RTSR to patients aged 60 years or older with osteoarthritis and an intact rotator cuff, this study’s findings provide reassurance that RTSR is an acceptable alternative in this patient group. Further research with more comprehensive patient reported outcomes is required to fully evaluate the cost effectiveness of this procedure and to examine any functional differences.

**What is already known on this topic**

International use of reverse total shoulder replacement (RTSR) has increased rapidly over the past two decades
RTSR is now being used beyond its original pathology group across many surgical indications without high quality evidence
This treatment uncertainty has been identified as a key priority by national healthcare agencies, prompting funding of international surgical trials that will take several years to complete

**What this study adds**

This study’s findings support the use of RTSR and anatomical total shoulder replacement (TSR) for patients aged 60 years or older with osteoarthritis and intact rotator cuff tendons who are in need of elective shoulder replacement surgery
No differences in modelled lifetime healthcare costs were found between RTSR and TSR in this patient group
Despite the difference in risk of revision surgery over time (favouring RTSR in the first three years), no clinically important differences were found in long term revision surgery, reoperations within 12 months, serious adverse events, or prolonged hospital stay

## Data Availability

Access to the data analysed in this study required permission from the National Joint Registry research subcommittee. Information on research data access requests to the National Joint Registry is available at https://www.njrcentre.org.uk/research/research-requests/. No additional data available.

## Web extra

Extra material supplied by authors

Web appendix: Supplementary appendix

## Notes

Transparency: The lead author (the manuscript’s guarantor) affirms that the manuscript is an honest, accurate, and transparent account of the study being reported; that no important aspects of the study have been omitted; and that any discrepancies from the study as planned (and, if relevant, registered) have been explained.

Dissemination to participants and related patient and public communities: The NJR and its patient representative group have been made aware of the results of this study and will support the dissemination of results through their networks. Dissemination to the wider public will be supported by the University of Oxford and the Oxford National Institute for Health and Care Research (NIHR) Biomedical Research Centre (BRC) through the press, media and charities. Surgeons and policy makers will be informed of the results through presentation at national and international scientific meetings.

Provenance and peer review: Not commissioned; externally peer reviewed
