# Supplementary material for: Reverse total shoulder replacement versus anatomical total shoulder replacement for osteoarthritis: population based cohort study using data from the National Joint Registry and Hospital Episode Statistics for England
Source: BMJ. 2024 Apr 30;385:e077939. doi: 10.1136/bmj-2023-077939 (PMC11058468; doi:10.1136/bmj-2023-077939)
Supplement: Supplementary file 1 — Web appendix: Supplementary appendix [file vale077939.ww.pdf]

Supplementary appendix

Table S1: OPCS-4 codes to identify reoperations 2
Table S2: ICD-10 codes to identify serious adverse events 3
Table S3: ICD-10 codes to identify comorbidities 3
Negative control outcomes 3
Figure S1: Relative and absolute risk for negative control outcomes 4
Table S4: Covariate balance for sensitivity analyses 5
Treatment effects for sensitivity analyses 7
Figure S2: Revision: sensitivity 1- high volume surgeons 7
Figure S3: Revision: sensitivity 2- balanced practice surgeons 8
Figure S4: Relative and absolute risk for secondary outcomes- sensitivity analyses 9
Parametric model parameters 10
Figure S5: Sensitivity analysis using FPSM models versus base case (Weibull) 10
Matched cohort 11
Figure S6: Mortality (matched) 11
Table S5: Model fit statistics for different distributions of mortality (matched) 11
Figure S7: Revision (matched) 12
Table S6: Model fit statistics for different distributions of revision (TSR) (matched) 12
Table S7: Model fit statistics for different distributions of revision (RTSR) (matched) 12
Weighted (IPTW) cohort 13
Figure S8: Mortality (IPTW) 13
Table S8: Model fit statistics for different distributions of mortality (IPTW) 13
Figure S9: Revision (IPTW) 14
Table S9: Model fit statistics for different distributions of mortality (TSR)(IPTW) 14
Table S10: Model fit statistics for different distributions of mortality (RTSR)(IPTW) 14
Hospital costs 15
Table S11: Hospital costs (matched) 15
Table S12: Hospital costs (IPTW) 15
Figure S10: Effect of discount rate 16
Oxford Shoulder Score (PROMS analysis) 17
Figure S11: Oxford Shoulder Score change histograms 17
Table S13: Oxford Shoulder Score change distributions 17
Table S14: Covariate balance for OSS responders vs non-responders 18
References 20

Table S1: OPCS-4 codes to identify reoperations

Procedure type	OPCS-4 codes	Anatomy codes
SAD/ACJ excision	O291	
	W572,W844	Z812,Z814,Z891
	T621,T622,T626,T628,T629	Z814,Z891
Rotator cuff repair	T791,T793,T794,T795	
	T641,T642,T643,T744,T648,T649,T67,T68	Z742
MUA +-release	W911,W913,W918,W919,W781,W784	Z813, Z814, Z891
	W911,W913, W918, W919, W781, W784	Z813, Z814, Z891
Washout/debridement	W80, W811, W812, W813, W815, W713	Z813, Z814, Z891
	Y223, Y311, Y318, Y319, Y321	Z813, Z814, Z891
	W18	Z691, Z692, Z693, Z694
Synovectomy	W691, W692, W693, T711	Z813, Z814, Z891
Osteomyelitis surgery	W18	Z691, Z692, Z693,Z694
Complex reconstruction	O108, O109	
Bone resection	W068, W069, W091, W092, W093, W094	Z691, Z692, Z693
	W095, W096, W097, W098, W099	Z691, Z692, Z693
Arthroscopy or other soft tissue	Y528,Y767	Z812, Z813, Z814, Z891
	W816,W817,W818,W819	Z812, Z813, Z814, Z891
	W83	Z812, Z813, Z814, Z891
	W843,W845,W846,W847,W848,W868, W869	Z812, Z813, Z814, Z891
	W881,W888,W889	Z812, Z813, Z814, Z891
	W891,W898,W899,O198,O199	Z812, Z813, Z814, Z891
	W714,W718,W719	Z812, Z813, Z814, Z891
	W694,W695,W698,W699,W711,W712	Z812, Z813, Z814, Z891
	T645,T651,T658,T659,T701,T702	Z812, Z813, Z814, Z891
	W562,W563,W564,W568,W569	Z812, Z813, Z814, Z891
Surgery for instability	O27	
	W77 (not W776), W841, W842	
	W72, W73, W74, W75	Z813, Z814, Z891
Reduction of dislocation	W652, W658, W689, W662, W668	Z813, Z814, Z891
	W669, W672, W674, W678, W679	Z813, Z814, Z891
Fixation of periprosthetic fracture	W19 (not W191), W20, W21, W22	Z691, Z692, Z693, Z813, Z814, Z891
	W23, W24 (not W241), W25, W26	Z691, Z692, Z693, Z813, Z814, Z891
	W651, W653, W654, W656, W661, W663, W664	Z691, Z692, Z693, Z813, Z814, Z891
	W671, W673, W677, W332	Z691, Z692, Z693, Z813, Z814, Z891
	O172, O173, O175, O178, O179	Z691, Z692, Z693, Z813, Z814, Z891

Table S2: ICD-10 codes to identify serious adverse events

Event	ICD-10 codes
Pulmonary embolism	I26
Myocardial infarction	I21,I22
Cerebrovascular event	I60,I61,I62,I63,I64
Acute kidney injury	N17
Lower respiratory tract infection	J12,J13,J14,J15,J16,J18,J22,J86,J440,J851,J690
Urinary tract infection	N10,N300,N308,N309,N390
Death	*Civil Registration Mortality data linked to NJR data*

Table S3: ICD-10 codes to identify comorbidities

Comorbidity	ICD-10 codes
Gastrointestinal diseases	K00-K93
Mental health diseases	F00-F99
Respiratory diseases	J00-J99
Circulatory diseases	I00-I99
Metabolic diseases	E00-E90 (not E66)
Neurological diseases	G00-G99
Urinary tract diseases	N00-N99
Health hazards	Z55-Z65
Obesity	E66

Negative control outcomes

Femur fracture ICD-10 codes: S71.x

Vertebral fracture ICD-10 codes: S12.x, S22.x, S32.x

Acute upper respiratory tract infection (URTI) ICD-10 codes: J00-J06

Hernia ICD-10 codes: K40-K46

Figure S1: Relative and absolute risk for negative control outcomes

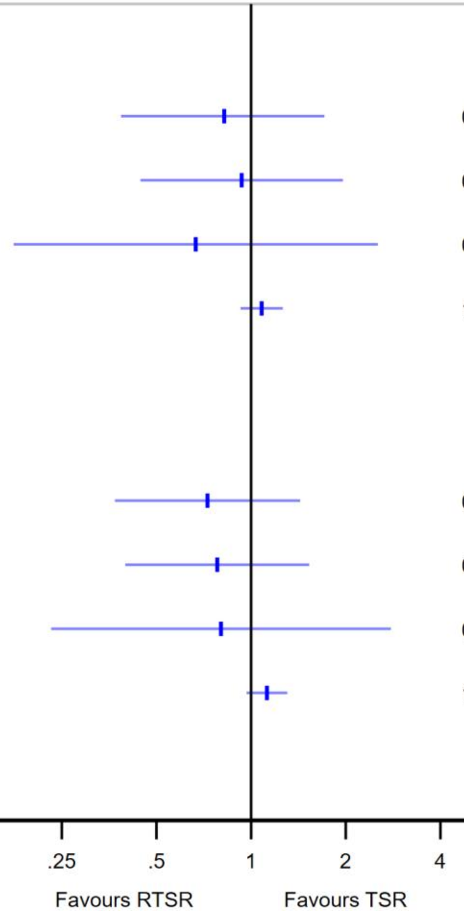

Subgroup and Outcome	Odds ratio (95% CI)	Events_TSR	Pop_TSR	Events_RTSR	Pop_RTSR	Absolute_risk_difference/% (95% CI)
**Base case - Matching**						
Femur fracture	0.82 (0.39, 1.71)	16	3562	13	3562	-0.08(-0.38,0.21)
Vertebral fracture	0.93 (0.44, 1.96)	16	3562	15	3562	-0.03(-0.33,0.28)
Acute URTI	0.67 (0.18, 2.53)	6	3562	4	3562	-0.06(-0.23,0.12)
Hernia	1.08 (0.93, 1.26)	340	3562	365	3562	0.70(-0.68,2.09)
**Base case - IPTW**						
Femur fracture	0.73 (0.37, 1.43)	30	9393	13	3575	-0.10(-0.30,0.10)
Vertebral fracture	0.78 (0.40, 1.53)	27	9393	15	3575	-0.08(-0.28,0.13)
Acute URTI	0.80 (0.23, 2.78)	8	9393	4	3575	-0.02(-0.11,0.08)
Hernia	1.12 (0.97, 1.30)	740	9393	367	3575	0.94(-0.29,2.18)

Table S4: Covariate balance for sensitivity analyses

	Sensitivity 1- High volume surgeons		Sensitivity 2- Balanced practice surgeons	
	Matched study population (n=5,044)	IPTW study population (n=8,627)	Matched study population (n=3,382)	IPTW study population (n=7,217)
**Characteristic**	**ASMD, %**	**ASMD, %**	**ASMD, %**	**ASMD, %**
Age	2.4	0.3	0.8	1.4
Sex				
Male	0.6	0.2	2.9	0.5
Female	0.6	0.2	2.9	0.5
ASA grade				
1	4.1	0.5	1.8	1.3
2	0.5	2.2	1.2	1.6
3	2.0	2.1	1.9	1.1
4/5	1.4	0.2	0.0	0.3
Operation funding				
Public (NHS)	1.9	0.7	1.1	1.7
Private	1.9	0.7	1.1	1.7
Previous surgery				
Yes	0.8	2.2	1.7	2.3
No	0.8	2.2	1.7	2.3
Thromboprophylaxis				
None	1.1	1.0	0.7	2.1
Chemical	1.2	0.1	2.3	2.0
Mechanical	1.1	2.6	2.0	1.9
Chemical & Mechanical	0.5	2.7	2.5	2.8
IMD decile				
1 (most deprived)	0.0	0.3	0.8	0.1
2	0.3	1.1	1.5	0.5
3	0.2	1.5	1.8	1.7
4	1.0	0.4	0.0	0.3
5	0.1	0.9	1.5	0.2
6	1.6	1.3	2.8	0.8
7	1.5	0.8	1.0	2.0
8	0.7	1.5	1.4	1.8
9	2.5	1.3	0.2	1.2
10 (least deprived)	0.7	1.6	4.1	5.3
Rural/urban residence				
Urban- sparse	1.8	0.1	0.0	0.4
Town and Fringe- sparse	2.3	1.2	2.5	3.2
Village- sparse	2.4	2.2	0.7	1.0
Hamlet- sparse	0.5	1.5	1.6	0.9
Urban- less sparse	0.6	0.1	0.9	3.5
Town and Fringe- less sparse	0.2	0.7	0.8	2.4
Village- less sparse	0.1	0.5	0.6	2.2

Hamlet- less sparse	0.2	0.5	1.9	0.6
Gastrointestinal diseases				
Yes	1.5	0.7	3.2	0.2
No	1.5	0.7	3.2	0.2
Mental health diseases				
Yes	1.5	0.5	2.1	0.8
No	1.5	0.5	2.1	0.8
Respiratory diseases				
Yes	0.5	0.6	0.9	0.2
No	0.5	0.6	0.9	0.2
Cardiac diseases				
Yes	1.7	1.4	0.1	0.7
No	1.7	1.4	0.1	0.7
Metabolic diseases				
Yes	0.6	0.7	0.2	2.5
No	0.6	0.7	0.2	2.5
Neurological diseases				
Yes	1.5	0.7	0.5	0.6
No	1.5	0.7	0.5	0.6
Urinary tract diseases				
Yes	0.7	0.9	0.8	0.3
No	0.7	0.9	0.8	0.3
Health hazards				
Yes	1.1	0.0	1.1	0.5
No	1.1	0.0	1.1	0.5
Obesity				
Yes	0.3	1.3	2.0	3.0
No	0.3	1.3	2.0	3.0
Charlson comorbidity index				
0	1.2	0.9	2.2	2.4
1	0.1	0.3	0.3	0.5
2+	1.3	1.3	2.4	2.9

Treatment effects for sensitivity analyses

Figure S2: Revision: sensitivity 1- high volume surgeons

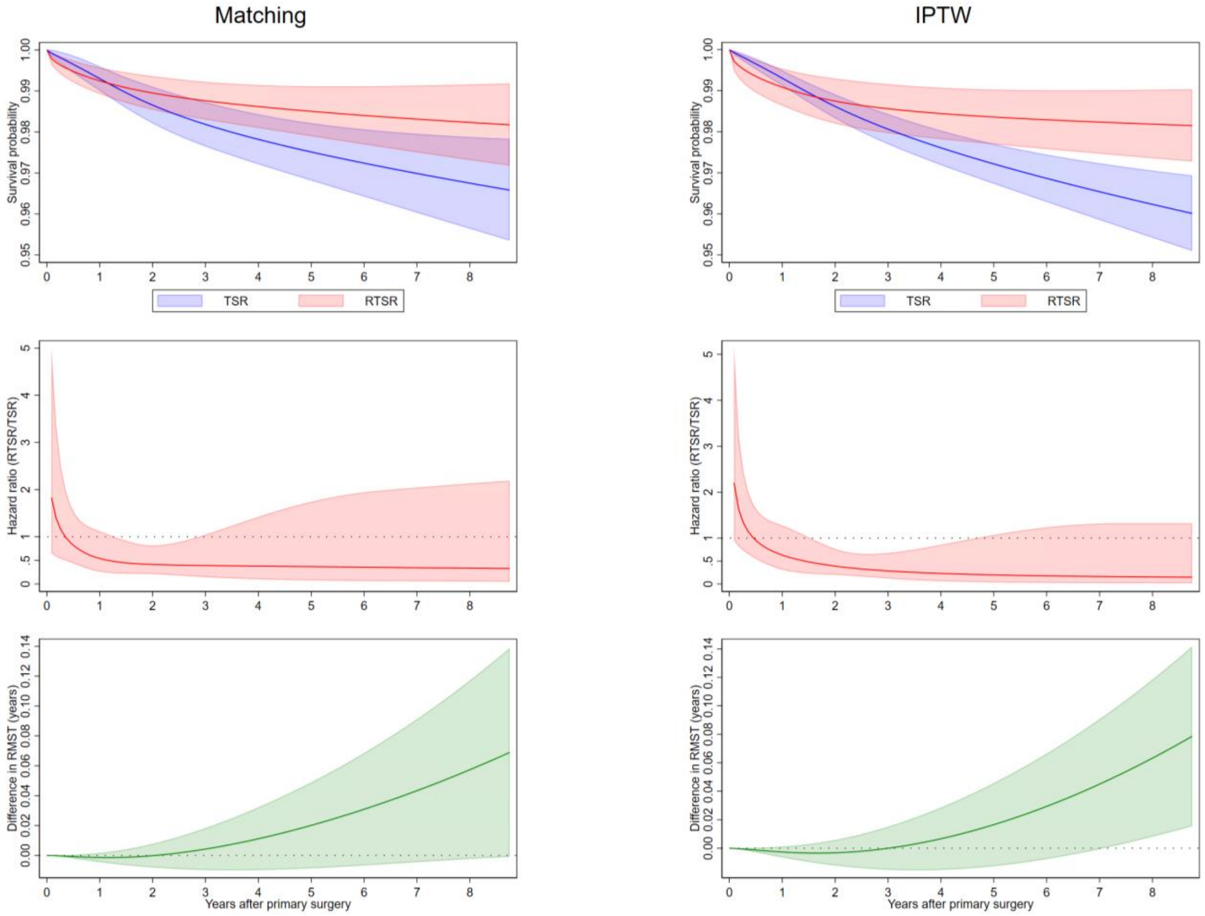

Figure S3: Revision: sensitivity 2- balanced practice surgeons

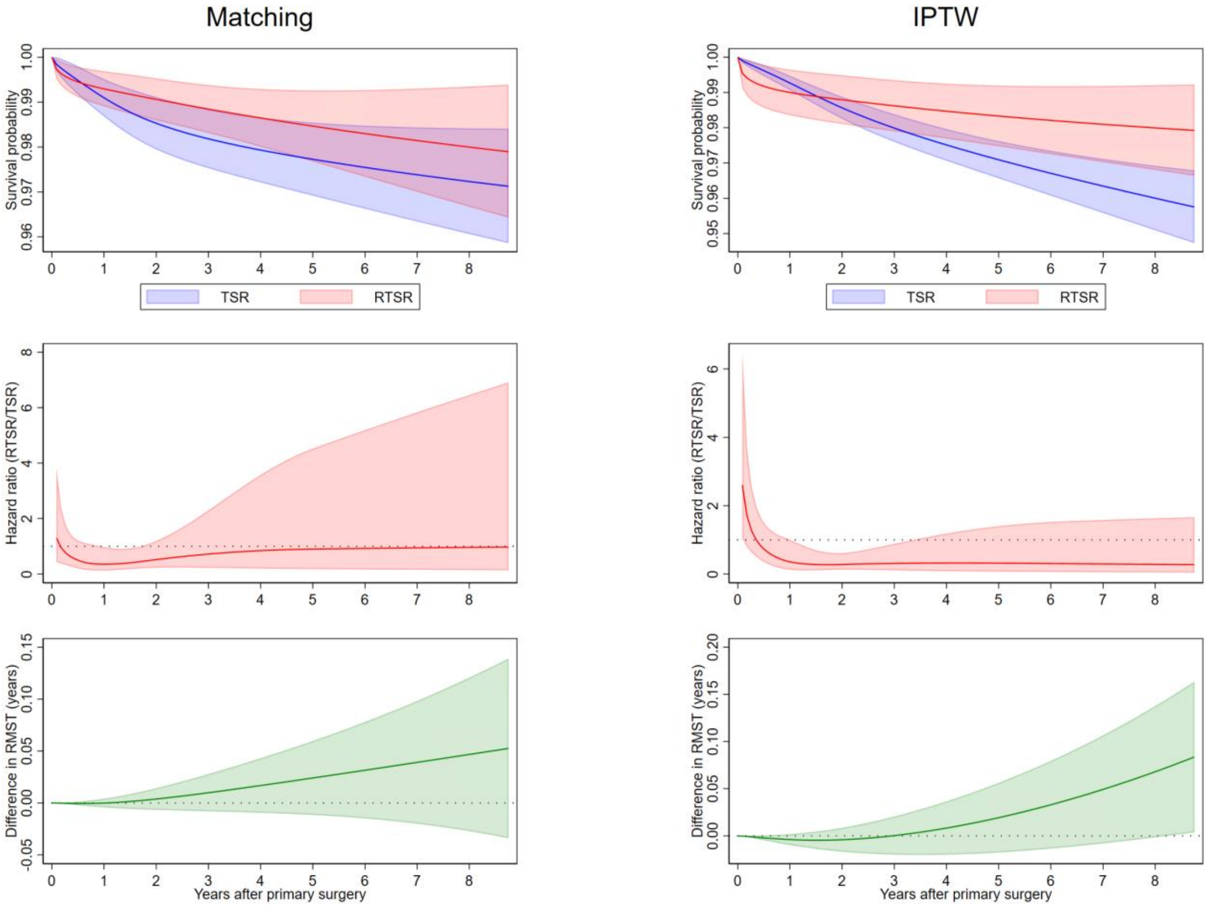

Figure S4: Relative and absolute risk for secondary outcomes- sensitivity analyses

Subgroup and Outcome	Odds ratio (95% CI)	Events_TSR	Pop_TSR	Events_RTSR	Pop_RTSR	Absolute_risk_difference/% (95% CI)
**Sensitivity 1 (High volume surgeons)- Matching**						
SAE 90-day	1.01 (0.78, 1.30)	124	2522	125	2522	0.04(-1.16,1.24)
Reoperations	0.65 (0.30, 1.40)	18	2522	12	2522	-0.24(-0.66,0.19)
Prolonged hospital stay	1.11 (0.96, 1.27)	478	2522	518	2522	1.59(-0.61,3.78)
**Sensitivity 1 (High volume surgeons)- IPTW**						
SAE 90-day	1.08 (0.84, 1.40)	203	6037	134	2590	0.32(-0.71,1.34)
Reoperations	0.64 (0.31, 1.33)	54	6037	12	2590	-0.30(-0.72,0.13)
Prolonged hospital stay	1.10 (0.96, 1.26)	785	6037	560	2590	1.24(-0.61,3.09)
**Sensitivity 2 (Balanced practice surgeons)- Matching**						
SAE 90-day	1.19 (0.88, 1.59)	86	1691	101	1691	0.89(-0.65,2.43)
Reoperations	0.38 (0.14, 1.08)	13	1691	5	1691	-0.47(-0.96,0.02)
Prolonged hospital stay	1.12 (0.95, 1.33)	365	1691	397	1691	1.89(-0.92,4.71)
**Sensitivity 2 (Balanced practice surgeons)- IPTW**						
SAE 90-day	1.26 (0.95, 1.69)	197	5524	102	1693	1.02(-0.32,2.36)
Reoperations	0.28 (0.10, 0.79)	53	5524	5	1693	-0.67(-1.04,-0.30)
Prolonged hospital stay	1.21 (1.02, 1.43)	745	5524	399	1693	2.66(0.22,5.09)

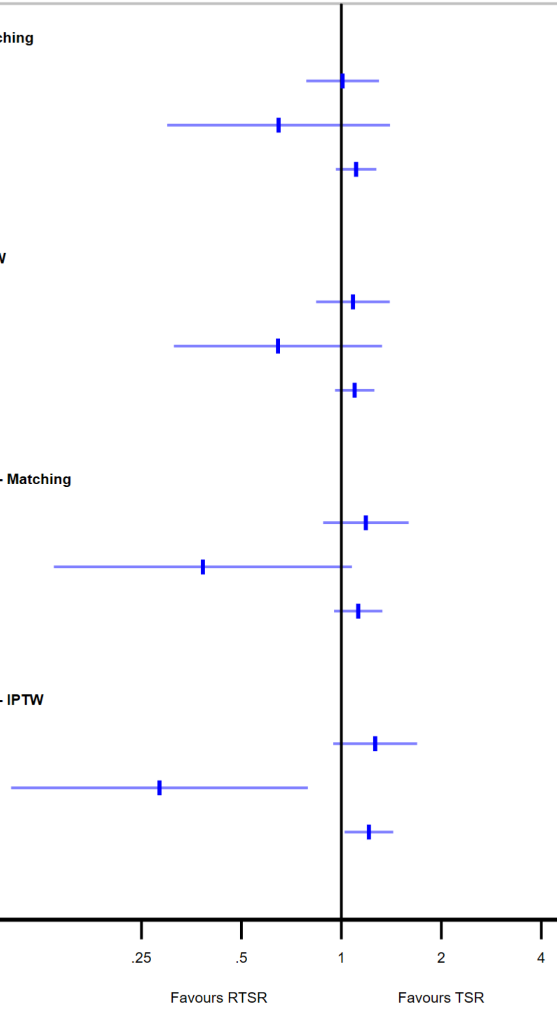

Parametric model parameters

Parametric model fit was assessed using the Akaike Information Criterion (AIC), Bayesian Information Criterion (BIC) and visual inspection, and the Weibull distribution fit best in all but one case where the log normal distribution was marginally better (TSR, revision, matched – Table S6). Given established methodological recommendation to use the same parametric distribution for different treatment arms unless there is strong evidence to suggest an alternative is more plausible, we used the Weibull distribution for both mortality and revision models for matched and weighted cohorts to inform transition probabilities for the cost analysis [1].

Flexible parametric survival models (FPSM) were used to model revision for the clinical effectiveness section of this study, and offered comparable model fit to the other parametric models used below. Given the necessity to extrapolate survival probabilities past the 8.75 years of follow-up, the more parsimonious parametric models were preferred over FPSM for the base case analysis. However, a sensitivity analysis was run using separate FPSM models for revision, yielding consistent results (Figure S5).

Figure S5: Sensitivity analysis using FPSM models versus base case (Weibull)

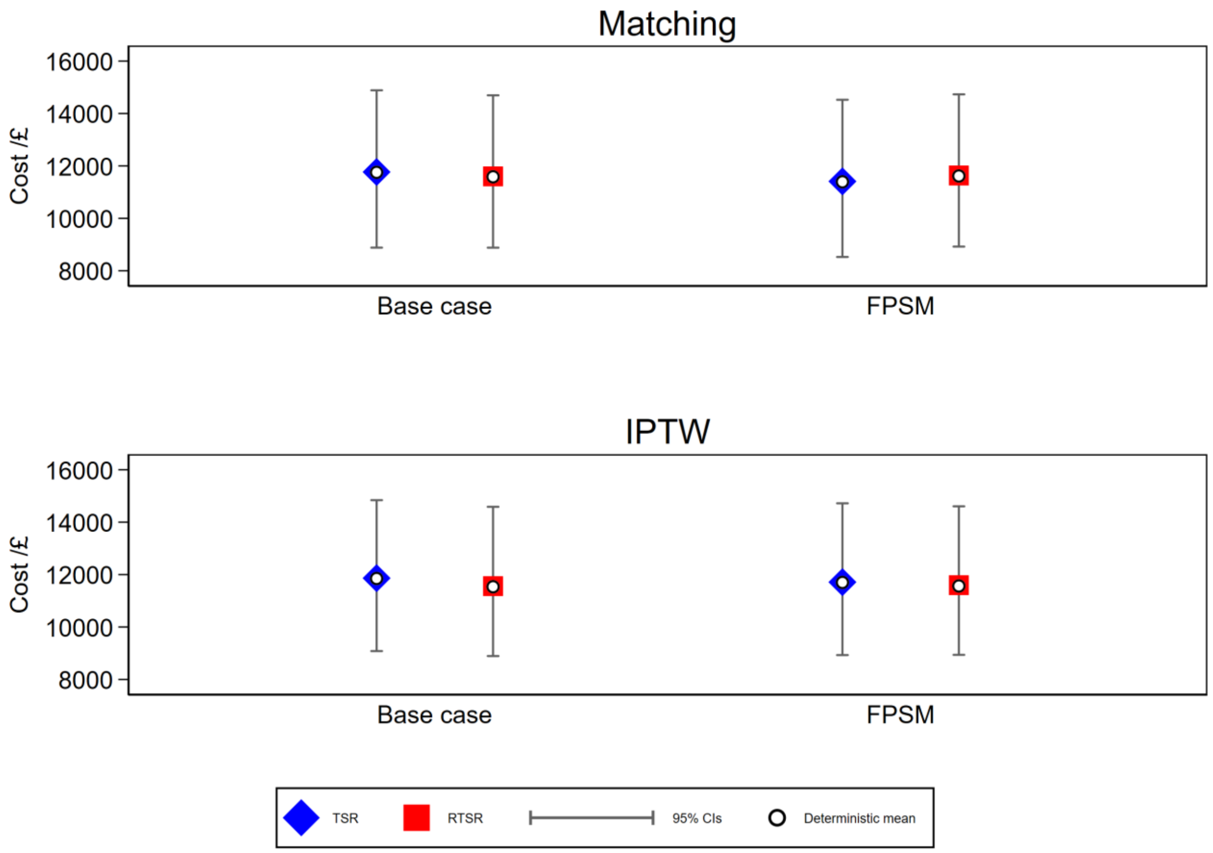

Matched cohort

Figure S6: Mortality (matched)

Hazards between TSR and RTSR groups were proportional (see log-log plot), so a single model was used with treatment group added as a covariate.

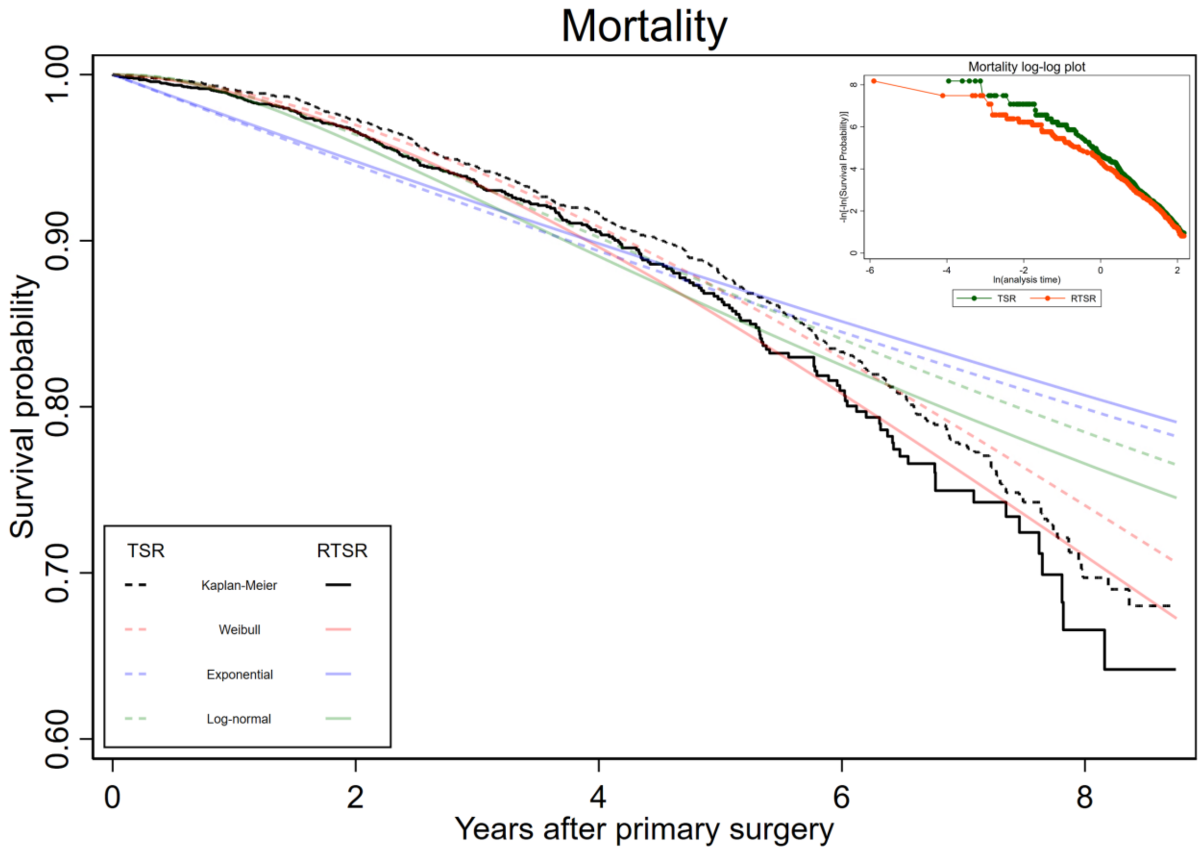

Table S5: Model fit statistics for different distributions of mortality (matched)

**Distribution**	**AIC**	**BIC**
Weibull	4729.0	4749.6
Exponential	4928.8	4942.5
Lognormal	4851.8	4872.5

Figure S7: Revision (matched)

Hazards between TSR and RTSR groups were not proportional, so separate models were used for each treatment group.

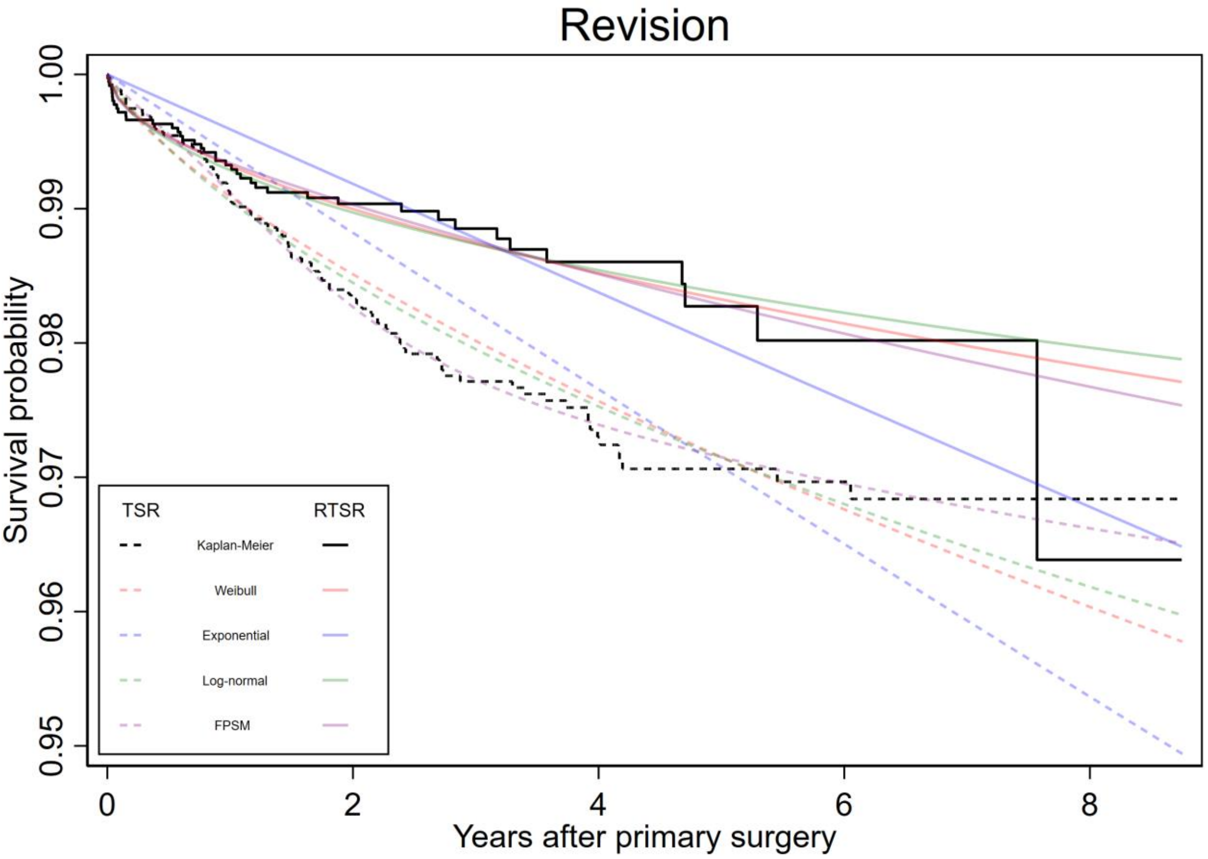

Table S6: Model fit statistics for different distributions of revision (TSR) (matched)

**TSR**		
**Distribution**	**AIC**	**BIC**
Weibull	1019.1	1031.5
Exponential	1029.7	1035.8
Lognormal	1016.4	1028.8
FPSM	1013.5	1038.2

Table S7: Model fit statistics for different distributions of revision (RTSR) (matched)

**RTSR**		
**Distribution**	**AIC**	**BIC**
Weibull	573.9	586.2
Exponential	591.4	597.6
Lognormal	574.3	586.7
FPSM	577.5	602.2

Weighted (IPTW) cohort

Figure S8: Mortality (IPTW)

Hazards between TSR and RTSR groups were proportional (see log-log plot), so a single model was used with treatment group added as a covariate.

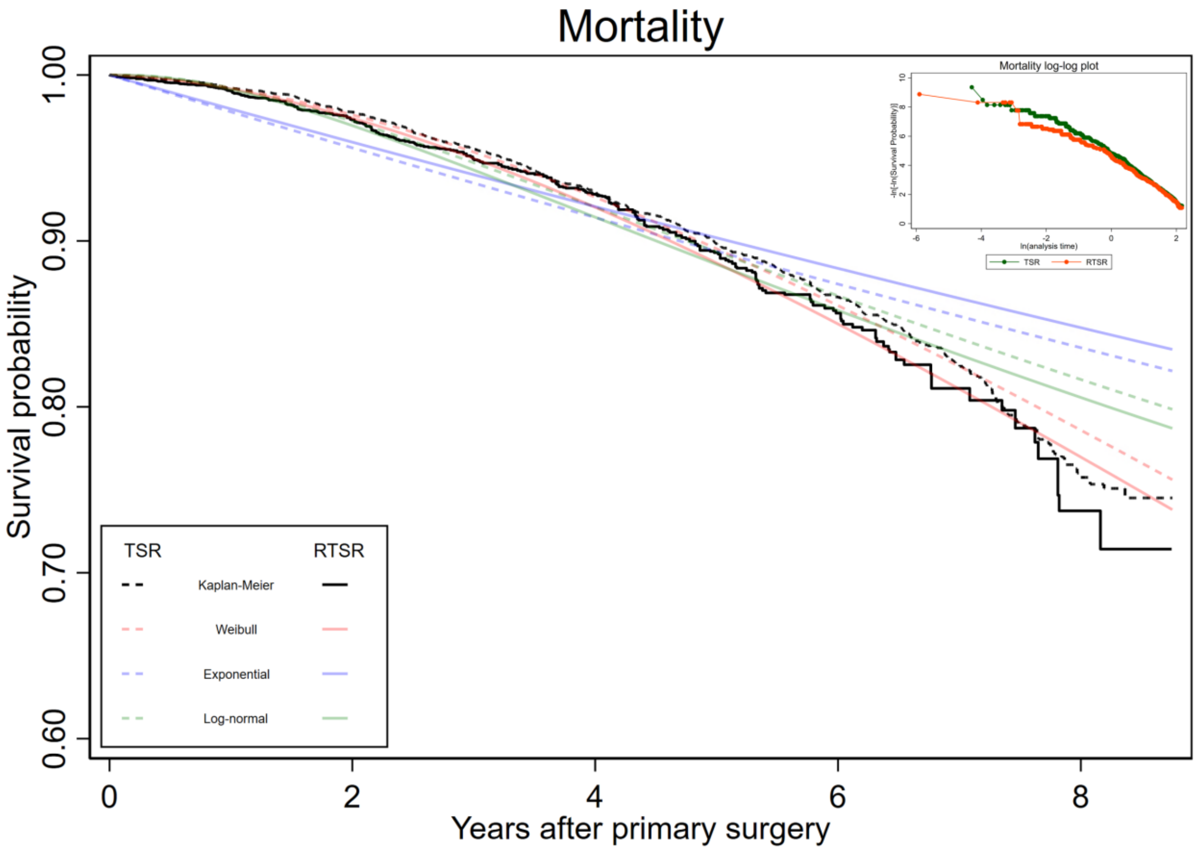

Table S8: Model fit statistics for different distributions of mortality (IPTW)

**Distribution**	**AIC**	**BIC**
Weibull	7826.6	7849.0
Exponential	8146.1	8161.0
Lognormal	7967.7	7990.1

Figure S9: Revision (IPTW)

Hazards between TSR and RTSR groups were not proportional, so separate models were used for each treatment group.

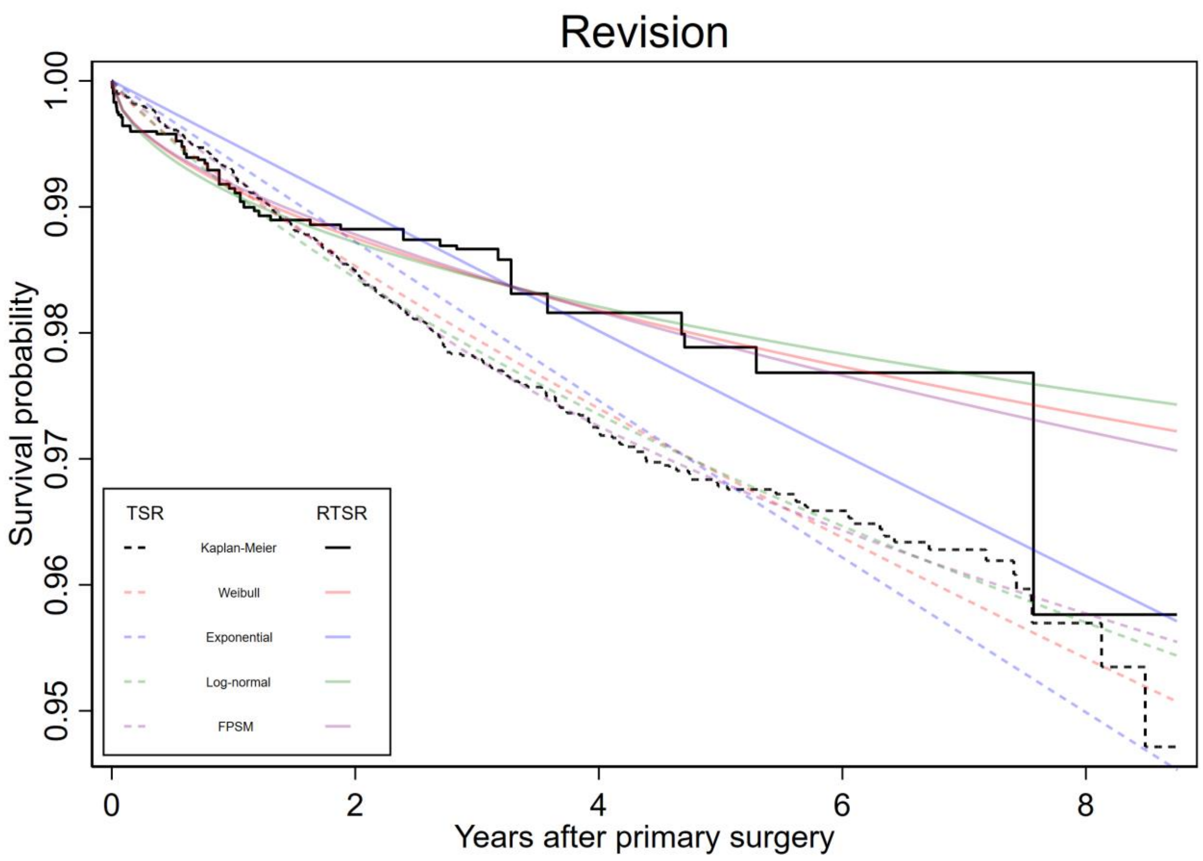

Table S9: Model fit statistics for different distributions of mortality (TSR)(IPTW)

TSR		
**Distribution**	**AIC**	**BIC**
Weibull	2778.0	2792.3
Exponential	2786.5	2793.6
Lognormal	2782.7	2797.0
FPSM	2772.7	2801.3

Table S10: Model fit statistics for different distributions of mortality (RTSR)(IPTW)

RTSR		
**Distribution**	**AIC**	**BIC**
Weibull	700.2	712.6
Exponential	725.5	731.7
Lognormal	701.3	713.7
FPSM	704.0	728.7

Hospital costs

Primary costs (sum of index primary procedure + SAE within 90 days + reoperations within 12 months).

Revision costs (sum of revision procedure + SAE within 90 days).

Distribution of actual costs and gamma (α, β) distributions for the base case analysis specified below.

Table S11: Hospital costs (matched)

	Primary				Revision			
	Mean (£)	Variance (£)	α	β	Mean (£)	Variance (£)	α	β
TSR	11140.09	2314501	53.61916	207.76322	13343.52	8164504	21.80776	611.8703
RTSR	11292.14	2251199	56.64201	199.3598	12614.00	11418705	13.93442	905.2406

Table S12: Hospital costs (IPTW)

	Primary				Revision			
	Mean (£)	Variance (£)	α	β	Mean (£)	Variance (£)	α	β
TSR	11060.79	2112864	57.90296	191.02288	13220.88	7993874	21.8657	604.6401
RTSR	11165.48	2132396	58.46379	190.9811	12535.44	11130857.7	14.11726	887.9511

Figure S10: Effect of discount rate

While the National Institute for Health and Care Excellence (NICE) recommends a discount rate of 3.5% used for the base case analysis, the below graph (representing the matched population) represents the effect of varying the discount rate, showing the results are robust to discount rates from 0 to 10% [2].

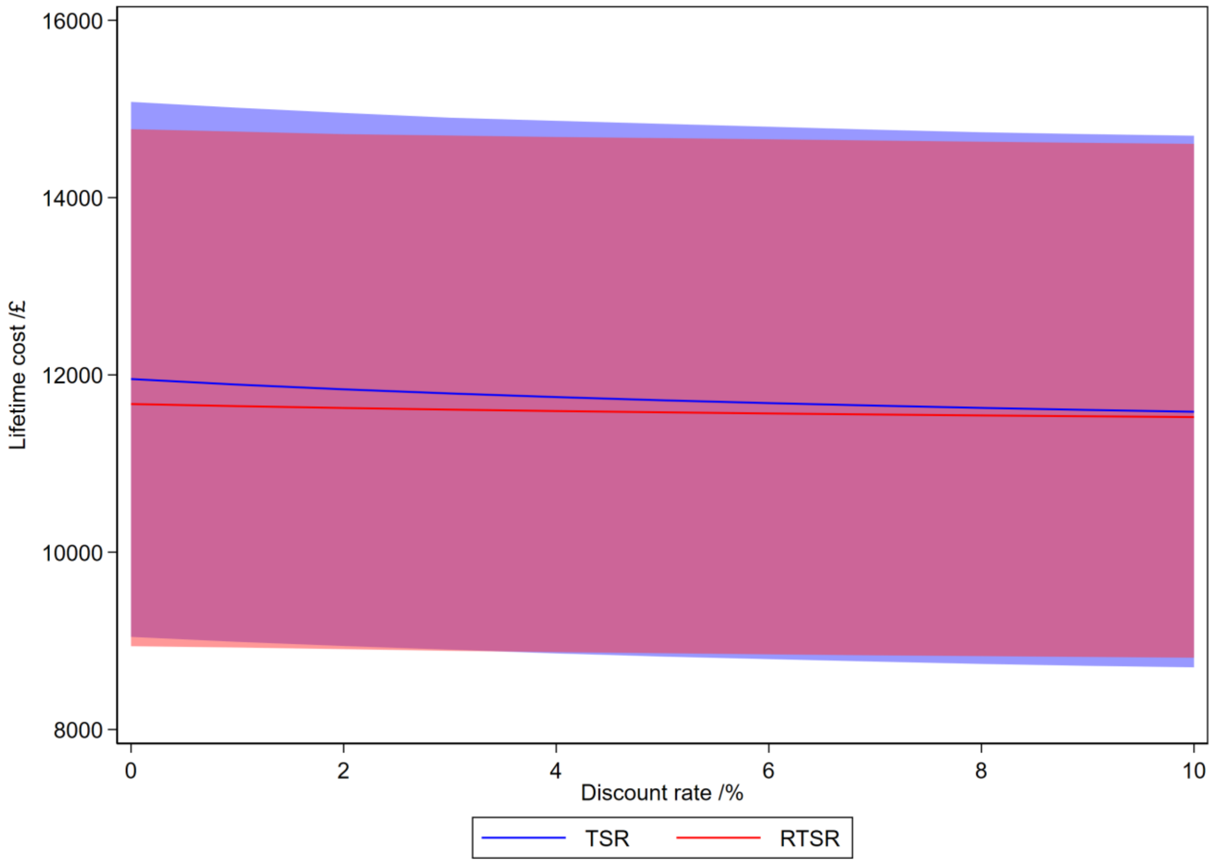

Oxford Shoulder Score (PROMS analysis)

Figure S11: Oxford Shoulder Score change histograms

Distributions of change in Oxford Shoulder Score [(6-month postoperative) – (preoperative)] for subset of non-missing change scores within base case matched and weighted (IPTW) populations.

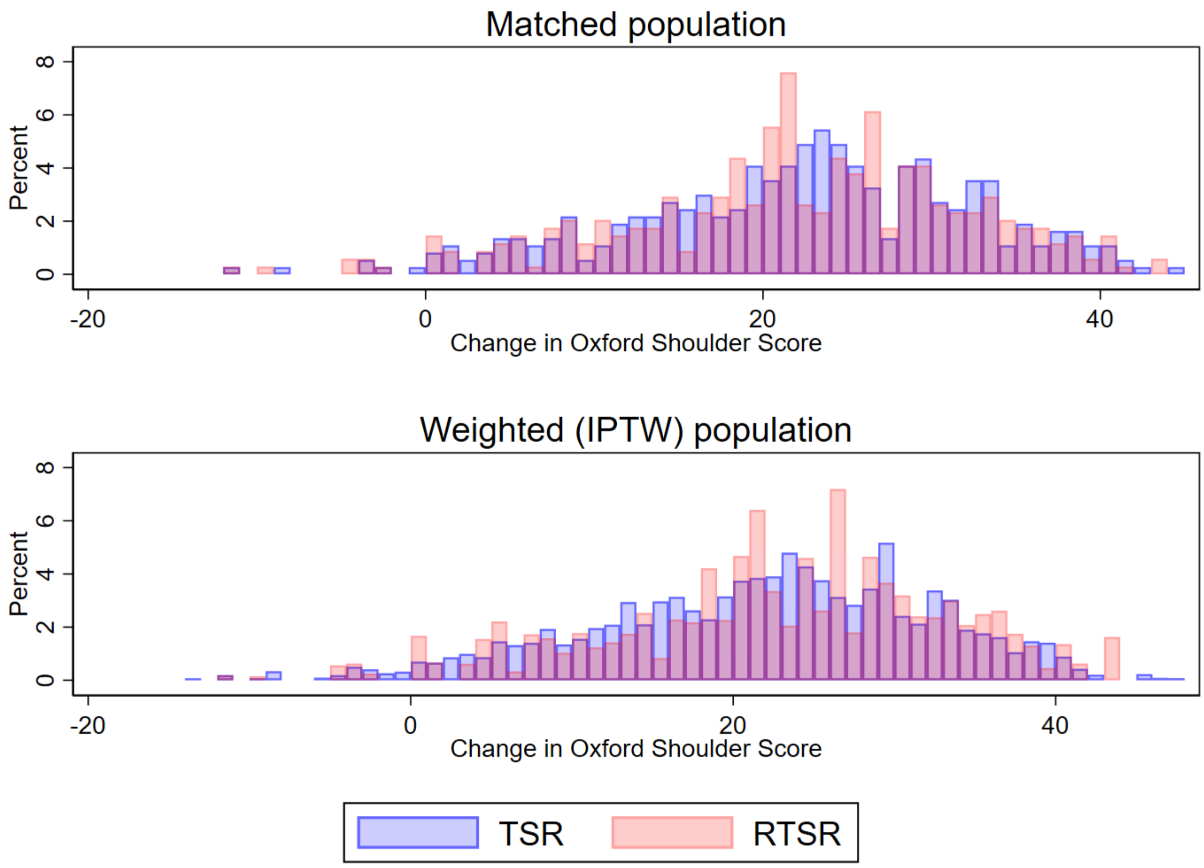

Table S13: Oxford Shoulder Score change distributions

	Matched population		Weighted population	
	TSR	RTSR	TSR	RTSR
Mean	21.8	21.6	21.4	22.4
SD	10.1	10.2	10.4	10.5

Table S14: Covariate balance for Oxford Shoulder Score responders vs non-responders

The below table shows the covariate balance in the matched and weighted cohorts, between patients who had a non-missing (responders) and missing (non-responders) Oxford Shoulder Score. The majority of covariates were well balanced with ASMD less than 10%, but there were a couple of categories of certain variables that had a slightly higher ASMD suggesting some minor imbalance.

	Matched study population (n=709 responders, n=6,415 non-responders)	IPTW study population (n=1,319 responders, n=11,649 non-responders)
**Characteristic**	**ASMD, %**	**ASMD, %**
Age	1.9	2.6
Sex		
Male	0.3	1.7
Female	0.3	1.7
ASA grade		
1	2.2	2.2
2	0.9	4.0
3	1.2	3.8
4/5	6.4	4.2
Operation funding		
Public (NHS)	4.2	8.8
Private	4.2	8.8
Previous surgery		
Yes	3.0	1.4
No	3.0	1.4
Thromboprophylaxis		
None	10.5	9.7
Chemical	10.2	8.9
Mechanical	7.4	7.7
Chemical & Mechanical	2.1	3.4
IMD decile		
1 (most deprived)	8.3	10.5
2	2.7	2.1
3	8.2	5.6
4	9.8	6.6
5	1.6	0.4
6	1.5	1.1
7	1.0	0.2
8	0.7	0.5
9	8.1	4.5
10 (least deprived)	10.7	13.3
Rural/urban residence		
Urban- sparse	4.8	3.4
Town and Fringe- sparse	4.7	2.0
Village- sparse	0.9	2.1
Hamlet- sparse	9.5	5.7
Urban- less sparse	8.9	5.5

Town and Fringe- less sparse	12.8	8.9
Village- less sparse	2.1	1.4
Hamlet- less sparse	0.0	0.1
Gastrointestinal diseases		
Yes	1.2	3.6
No	1.2	3.6
Mental health diseases		
Yes	1.2	1.3
No	1.2	1.3
Respiratory diseases		
Yes	0.3	1.3
No	0.3	1.3
Cardiac diseases		
Yes	4.8	0.4
No	4.8	0.4
Metabolic diseases		
Yes	2.4	4.6
No	2.4	4.6
Neurological diseases		
Yes	1.3	2.3
No	1.3	2.3
Urinary tract diseases		
Yes	3.8	5.6
No	3.8	5.6
Health hazards		
Yes	1.2	0.2
No	1.2	0.2
Obesity		
Yes	2.6	6.2
No	2.6	6.2
Charlson comorbidity index		
0	1.1	3.2
1	0.9	1.0
2+	1.9	2.5

References

1. Latimer N. *NICE DSU TECHNICAL SUPPORT DOCUMENT 14: SURVIVAL ANALYSIS FOR ECONOMIC EVALUATIONS ALONGSIDE CLINICAL TRIALS - EXTRAPOLATION WITH PATIENT-LEVEL DATA*.; 2011. Accessed August 14, 2023. http://www.nicedsu.org.uk/

2. NICE health technology evaluations: the manual. *(NICE), National Institute for Health and Care Excellence*. Published online 2022. https://www.nice.org.uk/process/pmg36/chapter/introduction-to-health-technology-evaluation
